# Potential of Genomic Selection in Mass Selection Breeding of an Allogamous Crop: An Empirical Study to Increase Yield of Common Buckwheat

**DOI:** 10.3389/fpls.2018.00276

**Published:** 2018-03-21

**Authors:** Shiori Yabe, Takashi Hara, Mariko Ueno, Hiroyuki Enoki, Tatsuro Kimura, Satoru Nishimura, Yasuo Yasui, Ryo Ohsawa, Hiroyoshi Iwata

**Affiliations:** ^1^Graduate School of Agricultural and Life Sciences, University of Tokyo, Tokyo, Japan; ^2^Graduate School of Life and Environmental Sciences, University of Tsukuba, Tsukuba, Japan; ^3^Graduate School of Agriculture, Kyoto University, Kyoto, Japan; ^4^Biotechnology and Afforestation Laboratory, Agriculture & Biotechnology Business Division, Toyota Motor Corporation, Miyoshi, Japan; ^5^Information System Development Department, X-Frontier Division, Frontier Research Center, Toyota Motor Corporation, Nagoya, Japan

**Keywords:** genomic selection, common buckwheat, phenotypic selection, selection index, allogamous plant species

## Abstract

To evaluate the potential of genomic selection (GS), a selection experiment with GS and phenotypic selection (PS) was performed in an allogamous crop, common buckwheat (*Fagopyrum esculentum* Moench). To indirectly select for seed yield per unit area, which cannot be measured on a single-plant basis, a selection index was constructed from seven agro-morphological traits measurable on a single plant basis. Over 3 years, we performed two GS and one PS cycles per year for improvement in the selection index. In GS, a prediction model was updated every year on the basis of genotypes of 14,598–50,000 markers and phenotypes. Plants grown from seeds derived from a series of generations of GS and PS populations were evaluated for the traits in the selection index and other yield-related traits. GS resulted in a 20.9% increase and PS in a 15.0% increase in the selection index in comparison with the initial population. Although the level of linkage disequilibrium in the breeding population was low, the target trait was improved with GS. Traits with higher weights in the selection index were improved more than those with lower weights, especially when prediction accuracy was high. No trait changed in an unintended direction in either GS or PS. The accuracy of genomic prediction models built in the first cycle decreased in the later cycles because the genetic bottleneck through the selection cycles changed linkage disequilibrium patterns in the breeding population. The present study emphasizes the importance of updating models in GS and demonstrates the potential of GS in mass selection of allogamous crop species, and provided a pilot example of successful application of GS to plant breeding.

## Introduction

Genomic selection (GS; Meuwissen et al., 2001) is a promising breeding technology to increase crop production and to improve world food security (Tester and Langridge, 2010). In GS, genetic ability is predicted with a model trained with genome-wide marker genotype data and phenotype data from a training population. For the genetic improvement of complex traits controlled by a number of genes, GS is advantageous over conventional marker-assisted selection, which targets mainly the selection of a few major QTL (Bernardo, 2008; Heffner et al., 2009, 2010; Jannink et al., 2010; Rutkoski et al., 2011). GS was first introduced in dairy cattle breeding in the late 2000s and has dramatically increased genetic gain per unit time (García-Ruiz et al., 2016). Especially rapid genetic improvement was observed in difficult-to-improve low-heritability traits. In plant breeding, the potential of GS was empirically evaluated in maize (*Zea mays* L.) (Massman et al., 2013; Beyene et al., 2015), oat (*Avena sativa* L.) (Asoro et al., 2013), and wheat (*Triticum aestivum* L.) (Rutkoski et al., 2015).

The efficiency of GS is expected to differ greatly between plant species because of differences in reproduction and breeding strategies (Lin et al., 2014) and the genetic nature of target traits (e.g., heritability and the number of responsible genes) within species (Spindel et al., 2015, 2016). It is certain that GS will be able to accelerate breeding of various crops that have been neglected so far. To meet the recent demand for using indigenous crops, such as minor cereals (Hinterthuer, 2017), local vegetables (Cernansky, 2015), bioenergy crops (Allwright and Taylor, 2016), medicinal plants (Kulkarni et al., 2016), and forage crops (O'Mara, 2012; Kingston-Smith et al., 2013), the breeding of various crop species will be required (Abberton et al., 2016). GS is expected to improve mass selection of allogamous crops, to which many crops mentioned above belong, because it accelerates mass selection cycles (Yabe et al., 2013) and allows the selection of good paternal parents before pollination even for seed yield relating traits (Yabe et al., 2014b). However, the potential of GS for mass selection in allogamous crops has not been studied empirically.

Common buckwheat (*Fagopyrum esculentum* Moench; 2*n* = 2*x* = 16) is an allogamous crop with small plant size and a short generation time (2–3 months per generation). So far, the genetic improvement of common buckwheat has been hampered by complete outcross with a self-incompatibility system controlled by the *S*-locus (reviewed in Lewis and Jones, 1992). Although a draft genome sequence of common buckwheat is available (Yasui et al., 2016), breeding through the use of genomics has not been applied to this crop.

The present study used a scheme recommended in a previous simulation study (Yabe et al., 2014b) to evaluate the potential of GS in mass selection of common buckwheat in comparison with phenotypic selection (PS) in a 3-year experiment. Because GS can be performed more frequently (at least twice a year) using offseason nursery than PS, the contribution of breeding cycle acceleration with GS was evaluated. To improve seed yield per unit area, we used a selection index, in which the target trait is evaluated via modeling the relationship between the target trait and the other traits; this approach can simultaneously improve traits related to yield (Hazel, 1943; Falconer and Mackay, 1996). The selection index approach is also useful in improving traits that cannot be evaluated on a single-plant basis. We applied principal component regression (PCR) to find a regression equation that predicts a target trait that cannot be measured on a single-plant basis from secondary traits and used it as the selection index. PCR summarizes the correlative structure among the secondary traits as principal components and regresses the target trait on the components. We evaluated the selection accuracy and the response of the selection index and related traits to selection. The accuracy of prediction models was evaluated by tracking the pattern of linkage disequilibrium (LD), a major factor affecting the efficiency of GS (Hayes et al., 2009; de los Campos et al., 2013). On the basis of the results, we discuss factors essential for successful application of GS to mass selection of allogamous crop species.

## Materials and methods

### Base and initial populations for breeding experiment

**Figure 1:**
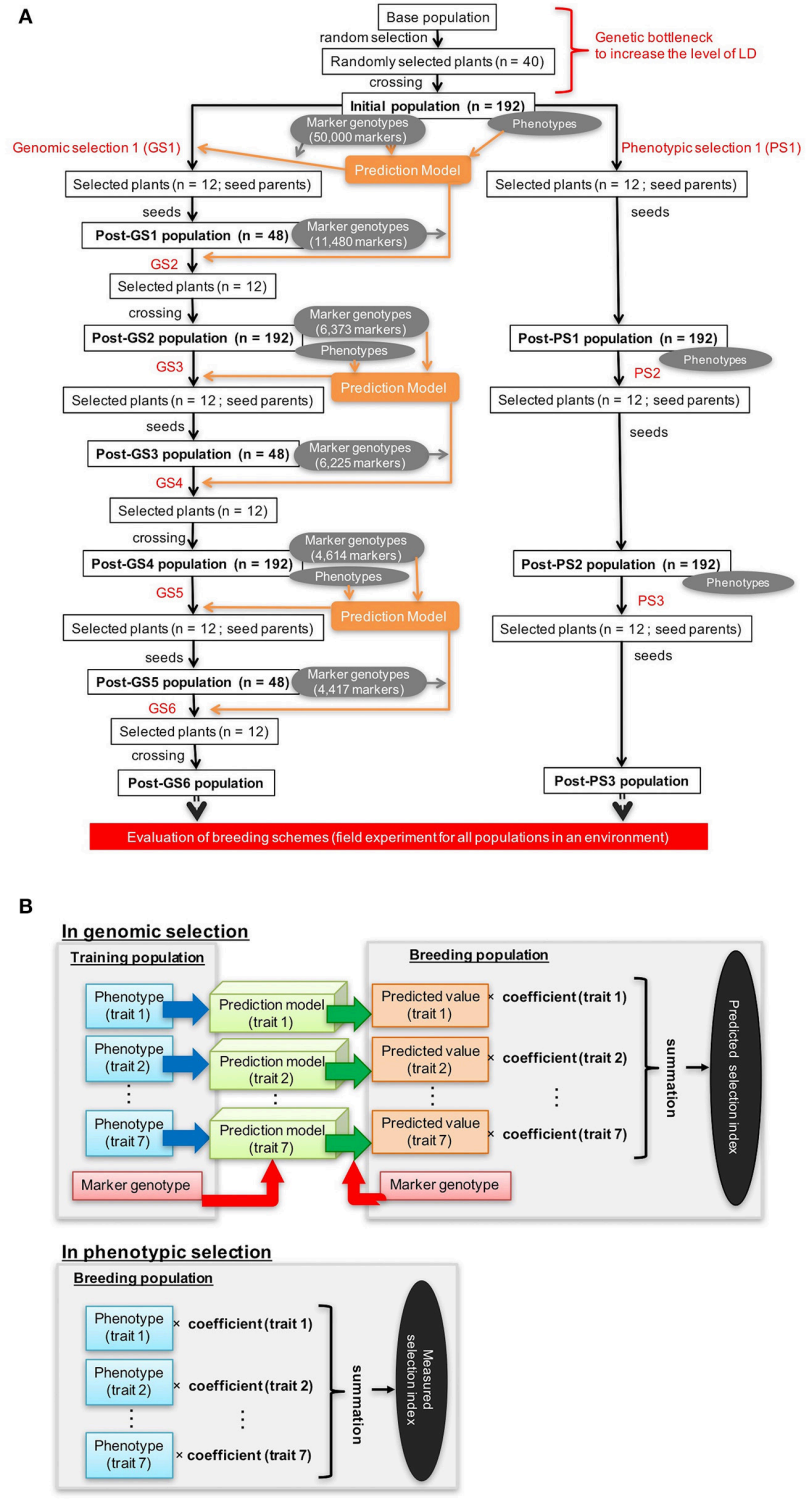
Genomic and phenotypic selection conducted in this study. **(A)** Scheme. **(B)** Calculation of the selection index.

The population 92FE1-F4 (Yabe et al., 2014a) was used as a base population. We expected that the outcrossing buckwheat population would have low LD (Nordborg, 2000; Flint-Garcia et al., 2003; Gupta et al., 2005), which would reduce the accuracy of GS. To increase LD in the initial population, we imposed a genetic bottleneck on the base population by performing one cycle of random mating with 40 plants. The progeny were used as the initial population for both GS and PS breeding (Figure 1A).

### Target traits and selection index

We built a selection index predictive of seed yield per unit area (kg/a) through modeling the relationship between yield and agro-morphological traits that can be measured in individual plants. The index was based on the data from a field experiment with 11 common buckwheat cultivars in Niigata Prefecture, Japan (38°18′N, 138°49′E), with a randomized block design with three replications. Seeds were sown (100 plants/m^2^; row length, 3 m; inter-row distance, 60 cm) on 19 May 1993. Fertilization with N:P:K = 0.4:1.6:1.6 kg/a was applied. We measured varietal averages of yield per unit area and seven traits: main stem length (cm), number of nodes, days to first flowering, number of flower clusters, number of primary branches, 1,000-seed weight (g/1,000 seeds), and test weight (g/l). PCR was used to build a regression model in which yield was a dependent variable and the seven traits were independent variables (in the R package “pls”; Mevik and Wehrens, 2007). The number of components was optimized via leave-one-out cross-validation to show the smallest root-mean-square error (RMSE).

The selection index of individual *i, s*_*i*_, was calculated as:

(1) $$s_{i}\;=\sum_{j}b_{j}u_{ij},$$

where *u*_*ij*_ represents the observed, expected, or predicted value of trait *j* of individual *i* respectively when we try to calculate observed, expected, and predicted selection index, and *b*_*j*_ is the regression coefficient of trait *j* derived from PCR.

### Genomic and phenotypic selection

GS and PS were performed for 3 years in an experimental field of the University of Tsukuba (36°06′N, 140°05′E) using potted plants in isolation cages (L × W × H, 630 × 540 × 230 cm) with honeybees as the pollinator. One plant was grown per pot. All pots (D × H, 24 × 24 cm), filled with normal culture soil, were placed randomly in each cage. The details are shown in Supplementary Table S1.

GS was conducted twice a year (Figure 1A). The first cycle in each year (GS1, GS3, and GS5 in Figure 1A) was performed in August. In this cycle, we performed the genotyping of the breeding population, plant crossing, phenotyping, model building or updating, and selection. As recommended (Yabe et al., 2013), we updated prediction models every year to take into account the pattern of LD change during selection. From the initial population (192 plants), 12 were selected in the basis of their expected selection index in GS1 (Figure 1A). For each trait included in the selection index, a prediction model was built with genomic best linear unbiased prediction (G-BLUP) (in R package “rrBLUP”; Endelman, 2011). The expected selection index values were calculated from the expected values of the seven traits with equation 1 (Figure 1B). Because all traits except days to first flowering were measured after pollination, plants were selected after pollination on the basis of the expected values of the selection index.

In the second GS cycle in each year (GS2, GS4, and GS6 in Figure 1A), we performed the genotyping of the breeding population, selection, and crossing. The second cycle was performed in an offseason nursery. Selection was on the basis of the predicted values of the selection index before pollination (because no trait evaluation was needed in this cycle). From 48 plants that were grown and genotyped, 12 were selected on the basis of their predicted selection index. A larger population in the first than in the second cycle was needed to train prediction models. To balance the genetic gain in whole selection cycles and the cost for genotyping, we set a different population size at the first and second cycles of each year.

We extracted total genomic DNA from the plants during the first and second cycles of each year and genotyped them as described by Yabe et al. (2014a). At GS1, we genotyped 274,303 candidate markers based on the raw sequencing reads using an Illumina Hiseq2000 (Illumina, Inc., San Diego, CA). We selected 50,000 markers according to their polymorphism, linkages with other markers, and clarity of the distinction between two genotypes in dominant markers (Yabe et al., 2014a). They were used them to build a prediction model. A microarray was developed using the sequencing data obtained at GS1 by the methods described by Yabe et al. (2014a). After GS1, the microarray markers were used for genotyping. To re-evaluate the quality of the markers, 12 plants genotyped at GS1 were genotyped again at GS2 with the 48 plants from the Post-GS1 population. The markers that were consistent between GS1 and GS2 were considered reliable. At GS2, 11,480 markers were selected on the basis of reliability (i.e., consistency of the marker genotypes between GS1 and GS2) in addition to the same marker selection way as GS1(according to their polymorphism and linkage with other markers). The original prediction model built at GS1 could not be used at GS2 because it was based on 50,000 markers that were not genotyped at GS2. Thus, at GS2, we rebuilt prediction models based on phenotypes and the data for 11,480 marker genotypes collected at GS1. After the first year, a total of 14,598 markers, which included 11,480 markers used at GS2, were used for genotyping. We used 6,373 markers at GS3, 6,225 at GS4, 4,614 at GS5, and 4,417 at GS6 for modeling; markers were selected as at GS2. The number of markers decreased because some markers became fixed and were excluded from the updated prediction models.

PS was conducted once a year from late July or early August to mid-October (Figure 1A; Supplementary Table S1). From the 192 plants of the breeding population, 12 were selected in each phenotyping round on the basis of their values of the selection index, which were calculated from the observed values of seven traits with equation 1 (Figure 1B). Because selection was conducted after pollination, selection was only imposed on the female parents.

### Evaluation of breeding schemes using phenotypes

Plants that emerged from seeds collected from the initial and all Post-GS and Post-PS populations were evaluated in 2013 and 2014 in the experimental field. One seed was sown per plastic pot (D × H, 24 × 24 cm) filled with normal culture soil as above; 48 seeds from each population were sown in 2013 and 36 seeds in 2014 (the initial population was not evaluated because of the insufficient number of seeds in 2013). All pots were placed randomly in an isolation cage (L × W × H, 1,500 × 720 × 450 cm). Nine traits (seven traits as above plus number of seeds per plant and number of secondary branches) were evaluated in both experiments. Population averages of these traits were compared by Welch's *t*-test with Bonferroni's correction.

### Evaluation of breeding schemes using marker genotypes

At GS1, GS3, and GS5, we performed leave-one-out cross-validation to evaluate the accuracy of GS, which was measured with Pearson's correlation coefficients between predicted and observed values.

The degree of LD, *r*^2^ (Hartl and Clark, 2007), was calculated for the initial population and Post-GS1 to Post-GS5 populations. The EM algorism was used to estimate *r*^2^ with dominant markers (Li et al., 2007). EM steps were repeated until the difference between consecutive estimated values became smaller than 0.0001. The methods proposed by Weir and Hill (1986) and Hill and Weir (1988) were used to estimate the expected (representative) *r*^2^ and the effective population size. Of 756 loci on the linkage map (Yabe et al., 2014a), 492 were polymorphic in the breeding population. The 492 mapped loci were represented by 1,511 markers genotyped in the breeding population (in some cases, several markers were located at the same locus), indicating that 10.4% of these markers were mapped on the linkage map (average interval between markers = 3.18 cM).

To evaluate deterioration in the accuracy of prediction models, models built at GS1 were applied to data collected at GS3 and GS5, and their accuracy was evaluated with Pearson's correlation coefficients.

## Results

### Construction of selection index

The coefficients of PCR were calculated with two principal components, in which the RMSE of prediction showed the minimum value, 1.3 (kg) in leave-one-out cross-validation. The calculated coefficients were used as the weight for each of the seven traits in the selection index (Table 1). The relationship among the seven traits used in the selection index was similar between the field experimental data used to build the selection index and the data for the initial breeding population (Table 2). The correlation between two correlation matrices (field experimental data and the initial population) was significant at the 5% level in the Mantel test.

**Table 1:** Principal component regression coefficients for traits used in the selection index.

**Trait**	**Coefficient**
Main stem length	+0.0550
Number of nodes	+0.0053
Days to first flowering	+0.0011
Number of flower clusters	+0.0728
Number of primary branches	+0.0015
1000-seed weight	−0.0001
Test weight	+0.0306

**Table 2:** Correlation coefficients among seed yield and seven traits used in the selection index.

	**Main stem length**	**Number of nodes**	**Days to first flowering**	**Number of flower clusters**	**Number of primary branches**	**1000-seed weight**	**Test weight**
Seed yield	0.74	0.85	0.05	0.66	0.52	−0.22	0.47
Main stem length		0.85	0.16	0.53	0.41	0.13	0.40
Number of nodes	0.56		0.12	0.75	0.64	−0.22	0.27
Days to first flowering	0.4	0.27		0.18	0.23	0.54	0.07
Number of flower clusters	0.36	0.46	0.26		0.73	−0.13	0.03
Number of primary branches	0.10	0.16	0.09	0.4		−0.37	−0.43
1000-seed weight	0.16	0.03	−0.04	0.04	−0.02		0.22
Test weight	0.13	0.26	0.09	0.31	0.19	0.24	

*Data from the field experiment used to construct the selection index are shown above the diagonal. Data for the initial breeding population are shown below the diagonal*.

### Genomic and phenotypic selection

**Figure 2:**
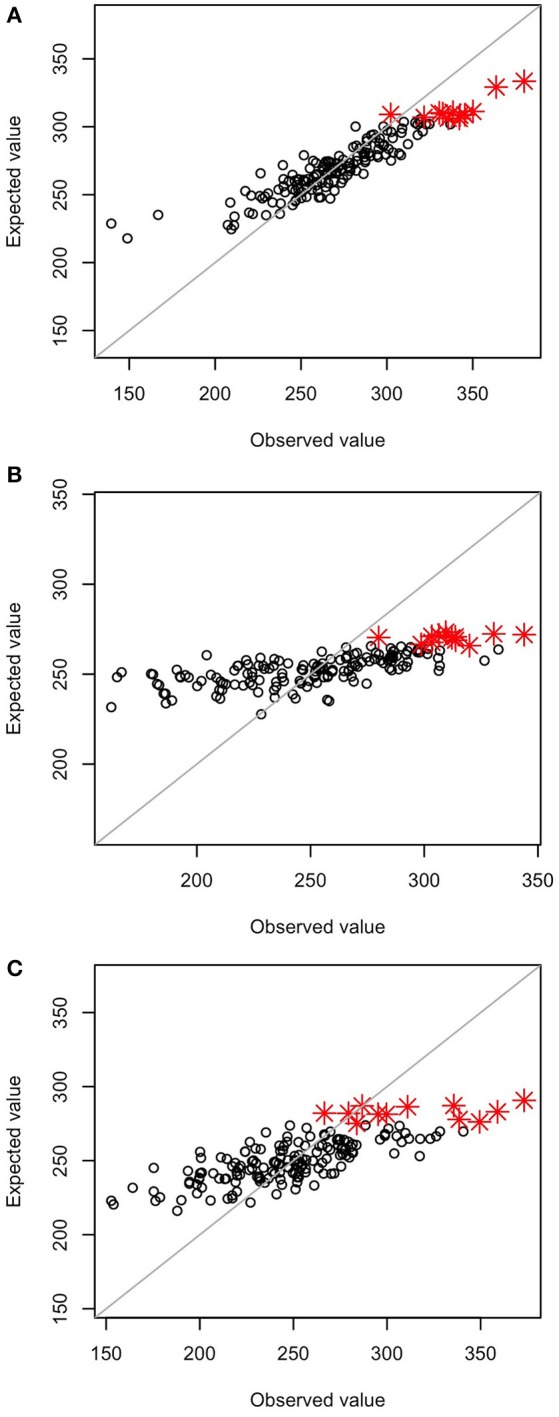
Relationship between observed values and values estimated by the prediction model. **(A)** GS1, **(B)** GS3, and **(C)** GS5. Red asterisks represent the 12 genotypes with the highest estimated values in the breeding population.

The correlation coefficients between observed and expected values of the selection index were 0.92 at GS1, 0.71 at GS3, and 0.77 at GS5; heavy shrinkage of expected values was observed at GS3 and GS5 but not at GS1 (Figure 2). GS1 and PS1 were conducted on the same initial population. Of the 12 plants with the highest index values at GS1, 9 were among the 12 plants selected at PS1. At GS1, however, the same 12 plants were selected at the time because of the miscalculation of the selection index. The miscalculation was caused by a programing error that selected a different marker set from the set selected as the microarray markers. However, the miscalculation did not influence the experiment because we selected the same 12 plants at GS1 and PS1. At GS6, 9 of the 12 selected plants died from disease, so 9 plants with the next highest index values were selected instead.

The prediction accuracy of the selection index was highest at GS1 and then decreased (Table 3). The accuracy for each of the seven traits included in the selection index also changed drastically. The accuracy for main stem length and the number of nodes sharply decreased in GS. The accuracy for the days to first flowering increased at GS3, and that for the number of flower clusters and 1,000-seed weight increased at GS5. The accuracy remained low throughout GS breeding for the number of primary branches and test weight. Even though test weight showed negative accuracy at GS3, we included it in the selection index because there was no large variation in its expected values; its inclusion did not affect plant rank according to the selection index.

**Table 3:** Correlation coefficients between predicted values (leave-one-out cross-validation) and observed values of seven traits and the selection index at GS1, GS3, and GS5.

	**GS1**	**GS3**	**GS5**
Main stem length	0.60	0.40	0.13
Number of nodes	0.53	0.41	0.27
Days to first flowering	0.27	0.53	0.52
Number of flower clusters	0.30	0.30	0.45
Number of primary branches	0.16	0.28	0.25
1000-seed weight	0.14	0.34	0.58
Test weight	0.26	−0.97	0.36
Selection index	0.46	0.10	0.23

**Figure 3:**
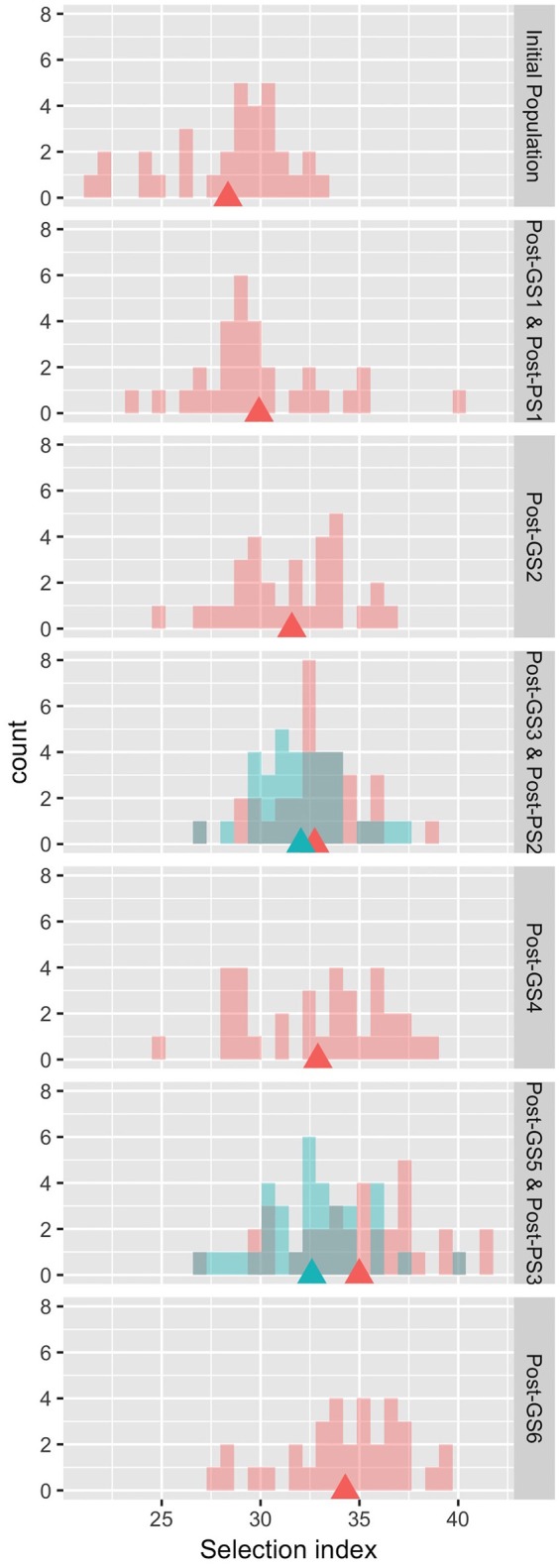
Distribution of the observed values of the selection index in the evaluation of breeding schemes in 2014. Rows 1, 3–7: pink, Initial and Post-GS populations; blue, Post-PS populations. Row 2: both populations are shown in pink. Triangles: population means.

The six GS cycles led to a 20.9% increase in the selection index from the initial population, whereas the three PS cycles led to a 15.0% increase (Table 4). In both GS and PS, two cycles of selection resulted in significant differences (*P* < 0.05) from the initial population, and the mean population value of the selection index gradually improved except at GS6, although the gain was not significantly different from zero in each cycle. The breeding population was improved as a whole (Figure 3). Although the Post-PS and Post-GS populations had different numbers of selection cycles, there was no large difference in the variance of the distribution of the selection index in any population. The agreement between the results obtained in 2013 (Supplementary Table S2) and 2014 (Table 4) suggests high repeatability of the experiment.

**Table 4:** Population means (and standard deviations) measured in 2014 to evaluate the breeding schemes after 3 years of selection.

	**Main stem length (cm)**	**Number of nodes**	**Days to first flowering (days)**	**Number of flower clusters**	**Number of primary branches**	**1,000-seed weight (g)**	**Test weight (g/l)**	**Number of secondary branches**	**Number of seeds in a plant**	**Selection index**
Initial population (*n* = 34)	100.50 (17.72) d	11.15 (1.44) d	20.41 (1.23) c	69.91 (23.78) c	4.44 (0.93) n.s.	29.27 (2.83) n.s.	565.92 (61.19) c	7.41 (2.91) n.s.	407.68 (181.60) c	28.35 (3.09) (100%) d
Post-GS1, Post-PS1 (*n* = 30)	112.2 (14.16) c	11.87 (1.50) cd	21.27 (0.98) bc	82.70 (29.07) bc	4.47 (0.97)	29.27 (3.10)	576.42 (47.24) bc	7.97 (2.30)	452.33 (173.99) abc	29.92 (3.34) (105.53%) cd
Post-GS2 (*n* = 31)	117.74 (15.28) bc	12.35 (1.30) bc	21.61 (1.56) ab	84.52 (25.19) bc	4.55 (0.99)	30.39 (3.79)	616.43 (46.43) ab	8.16 (2.63)	454.52 (137.11) bc	31.58 (2.81) (111.04%) bc
Post-PS2 (*n* = 34)	117.91 (14.63) bc	12.18 (1.29) bcd	21.15 (0.86) bc	90.12 (22.37) b	4.74 (0.96)	29.60 (2.92)	617.80 (26.58) a	8.03 (2.12)	524.94 (147.04) abc	32.04 (2.31) (113.02%) bc
Post-GS3 (*n* = 34)	125.29 (16.77) ab	12.47 (1.35) bc	21.91 (1.38) ab	101.29 (24.75) ab	4.82 (0.90)	28.37 (3.41)	600.67 (49.37) abc	9.00 (2.70)	562.24 (137.70) ab	32.74 (2.25) (115.48%) ab
Post-GS4 (*n* = 35)	133.14 (14.58) a	12.94 (1.28) ab	22.31 (1.43) a	100.94 (31.01) ab	4.60 (1.06)	30.28 (2.70)	593.85 (49.00) abc	8.23 (1.78)	539.00 (166.49) abc	32.90 (3.45) (116.05%) ab
Post-PS3 (*n* = 35)	119.17 (12.46) bc	12.06 (1.64) bcd	21.40 (2.74) abc	98.57 (34.04) ab	4.74 (1.15)	29.91 (3.82)	613.63 (51.47) ab	9.00 (2.39)	515.94 (115.81) abc	32.60 (2.74) (114.98%) ab
Post-GS5 (*n* = 35)	130.89 (17.82) ab	13.26 (1.46) ab	21.91 (1.29) ab	123.51 (35.42) a	4.31 (0.87)	29.49 (3.13)	611.20 (46.57) ab	9.37 (1.96)	556.40 (170.56) ab	34.99 (3.49) (123.42%) a
Post-GS6 (*n* = 33)	129.21 (16.37) ab	13.67 (1.55) a	22.06 (2.01) ab	114.42 (28.73) a	4.85 (0.83)	28.46 (2.93)	612.75 (44.11) ab	9.21 (2.52)	588.21 (167.63) a	34.29 (3.12) (120.94%) a

*The phenotypic values of populations marked with different letters were significantly different (P < 0.05); n.s., not significant. For the selection index, percentage values relative to the initial population (100%) are also shown*.

A significant gain in main stem length, number of flower clusters, and test weight was achieved in both GS and PS, and in the number of nodes, days to first flowering, and the number of seeds set per plant in GS (Table 4). GS1 (PS1) resulted in a significant increase in main stem length. No significant improvement was observed in the number of primary branches, 1,000-seed weight, or number of secondary branches during the 3 years. The patterns of the nine traits and the selection index were similar in 2013 and 2014 (Table 4, Supplementary Table S2).

### Ex-post analysis

**Figure 4:**
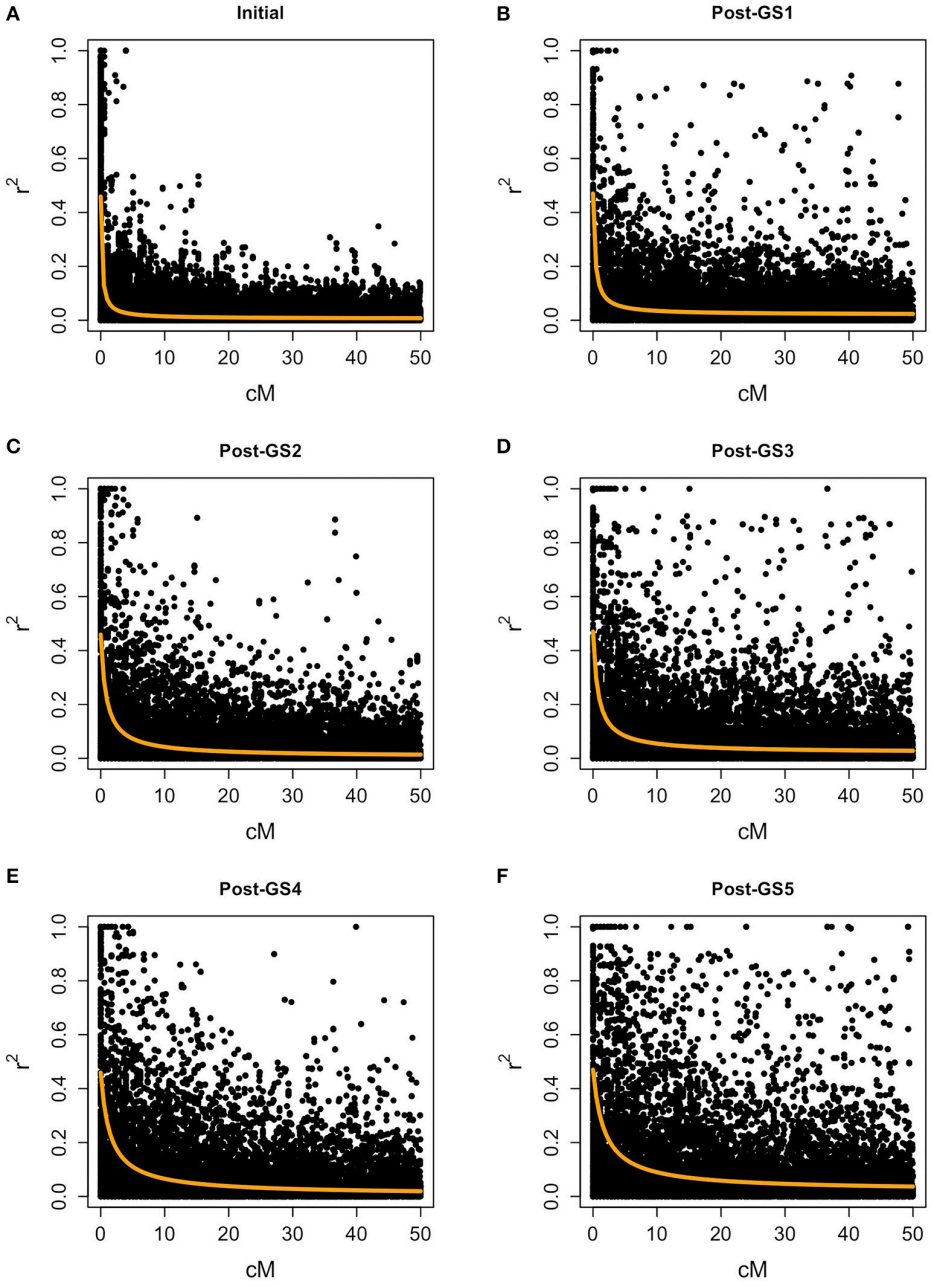
Linkage disequilibrium between polygenic markers within 50 cM on a chromosome. Populations: **(A)** Initial, **(B)** Post-GS1, **(C)** Post-GS2, **(D)** Post-GS3, **(E)** Post-GS4, **(F)** Post-GS5. Horizontal axes represent genetic distance between markers (cM). Orange lines show the expected *r*^2^.

In the initial population, the level of LD was low for most marker pairs, but high for some pairs of closely linked markers (Figure 4). The expected effective population size of the initial population (279.48) was large. LD increased with selection cycles, and the expected effective population size decreased rapidly (165.20 in the Post-GS1 population, 60.99 in Post-GS2, 65.58 in Post-GS3, 35.11 in Post-GS4, and 29.17 in Post-GS5).

**Figure 5:**
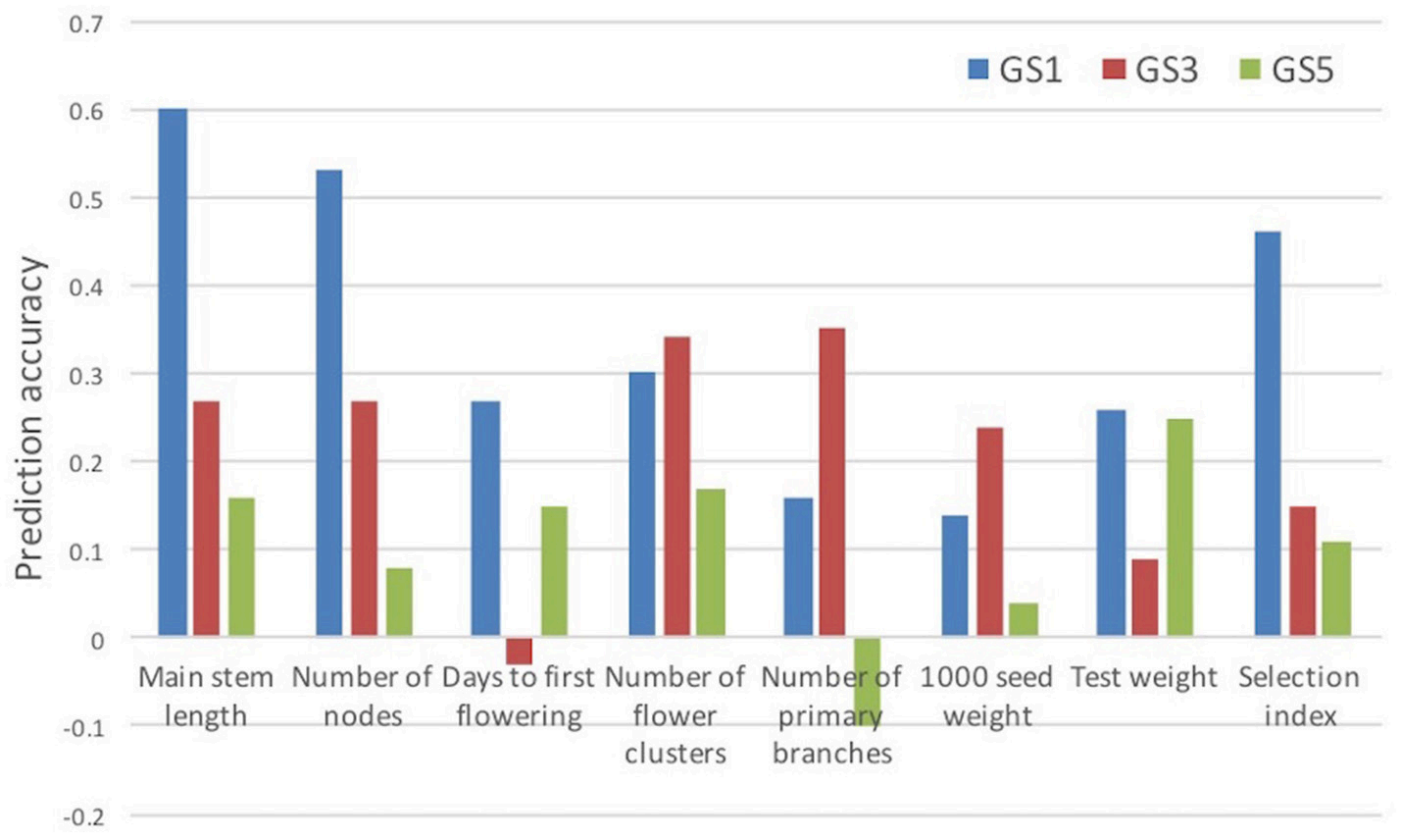
Prediction accuracy (correlation between estimated and observed values) for selection index and its seven component traits. Prediction models built at GS1 were used.

We evaluated the accuracy of prediction models built at GS1 for traits measured at GS3 and GS5. The accuracy was lower at GS3 than at GS1 for four traits and the selection index, and at GS5 for all traits and the selection index (Figure 5). The accuracy was lower at GS5 than at GS3 for five traits and the selection index. We found that only one plant was common among the 12 plants selected at GS3 and the 12 plants selected on the basis of the models built at GS1 (Supplementary Figure S1). This low commonality suggests that different plants would have been selected and that the genetic composition of the breeding population would have been different if we had not updated the prediction models.

## Discussion

In this study, GS improved the selection index that was constructed to represent seed yield per unit area. In 3 years, the mean selection index increased by 20.9% from that of the initial population in GS breeding and by 15.0% in PS breeding (Table 4). Plants requiring long terms for flowering show high seed yield in many crops (e.g., rice; Crowell et al., 2016). In our Post-GS6 population, days to first flowering was delayed in comparison with that of the initial population, which belong to “summer” agro-ecotype,” by 1.65 days on average. This just small delay suggests that the Post-GS6 population has good potential to be a new intermediate “summer” agro-ecotype cultivar with the same yield and earlier flowering than intermediate “autumn” agro-ecotype cultivars. The goal of mass selection of allogamous crops is to obtain a population with better and more stable phenotypes (Bos and Caligari, 2008). In the present study, the average yield potential was improved through GS (Figure 3, Table 4), but the phenotypic variance of the selection index did not decrease in comparison with that of the initial population in spite of the genetic bottleneck during the six GS cycles.

Unlike in previous studies (e.g., Asoro et al., 2013; Rutkoski et al., 2015), no phenotypic data were available before the training of prediction models, because phenotypic data collected in the past could not be linked to the current genotypes owing to the allogamous characteristics of common buckwheat. The present study suggests that a rapid improvement of complex traits with GS can be attained when both phenotype and genome-wide marker data are available for the target breeding population.

The selection index was used as the selection criterion for improving seed yield per unit area (the target trait), which cannot be measured on a single-plant basis in completely outcrossing species without clonal propagation ability. The selection index was constructed by modeling the relationship between yield and other traits that were measurable on a single-plant basis (Table 1). The selection index was based on the data from a past field experiment with a small number of cultivars. We found a similar among-trait correlation structure between the field experiment and the initial breeding population (Table 2), suggesting the applicability of the selection index to this population. The six GS cycles significantly increased the selection index (Table 4), although the prediction accuracy at GS3 and GS5 was low (Table 3). Expected values at these cycles were heavily shrunk to the overall average, and the improvement at each cycle was small, especially during the second year, in which the prediction accuracy of the selection index was the lowest among selection cycles (Table 3). The relation between prediction accuracy and selection response suggested that the degree of improvement of the target trait depended mainly on its prediction accuracy. The realized response to selection at GS4 and GS6 was smaller than that at the other cycles (Figure 3). This may result from the low predictive accuracy at GS3 and GS5 and from deterioration in the accuracy of the prediction models due to the changes in the LD pattern, as discussed later. The values of three traits with relatively high weight in the index (main stem length, number of flower clusters, and test weight) significantly increased in both PS and GS (Tables 1, 4), suggesting that weight in the selection index worked as expected (i.e., the higher weight traits had, the more they would be improved) at each selection cycle. In particular, the large weight of main stem length in the index (Table 1) and high prediction accuracy at GS1 (Table 3) might have led to a significant difference between the initial and Post-GS1 populations (Table 4). GS did not change the number of primary branches, probably because of its small weight in the index and low prediction accuracy throughout the selection cycles. In our study, the prediction accuracy decreased for some traits but increased for others. The response of each trait to selection (Falconer and Mackay, 1996) was automatically balanced so that a moderate improvement of the selection index was maintained during the six GS cycles. No severe trade-off relationship among traits was observed in either GS or PS (Table 4), showing that the selection index overcame the common trade-off relationship between the number of seeds set per plant and 1,000-seed weight (Gambín and Borrás, 2010), which limits genetic improvement of yield owing to the difficulty of selection for multiple traits that are negatively correlated with yield (Casler and Brummer, 2008), and the risk of a change in traits other than the target trait via linkage drag in GS (Asoro et al., 2013; Rutkoski et al., 2015). Massman et al. (2013) showed that the improvement in the selection index was not accompanied by an improvement in each trait included in it. The results of the above studies and our results suggest that the use of the selection index may balance different traits. The selection index could associate seed yield per unit area with marker genotypes and help to improve multiple traits simultaneously.

In common buckwheat and other allogamous species, a low LD level is a crucial issue for GS breeding because it decreases prediction accuracy; a strategy to overcome this issue is needed (Hayes et al., 2013; Lin et al., 2014; Resende et al., 2014). Previous studies of GS used elite populations, which were expected to have high levels of LD because of their small effective size. For example, GS in maize showed high LD between adjacent markers (i.e., *r*^2^ = 0.45) even with 287 SNPs (Massman et al., 2013). In the present study, although the genetic bottleneck was applied to the initial population (Figure 1), the expected LD values were quite low (e.g., *r*^2^ = 0.08 between markers 1 cM apart in the initial population and 0.13 in the Post-GS1 population; Figure 4), lower than the required *r*^2^-value (0.2; Hayes et al., 2009). However, GS breeding worked well (Table 4, Figure 3), with 0.46 of prediction accuracy at GS1 (i.e., selection for the population with quite low LD) (Table 3), probably because of the use of a prediction model based on G-BLUP, which captures relationships between individuals based on marker genotypes. A certain level of prediction accuracy can be attained with G-BLUP even when a quantitative trait locus (QTL) and markers are in linkage equilibrium (Habier et al., 2007). de los Campos et al. (2013) showed that the marker-derived realized relationship between selection candidates and individuals in a training population could represent the QTL-derived relationship when they were closely related. In the present study, the prediction model could capture the genomic relationship because the training population was closely related to or identical to a population of selection candidates. To preserve the accuracy under repeated selection cycles, LD between a QTL and markers should be constant during the cycles (Habier et al., 2007; Liu et al., 2015). When the pattern of LD in a breeding population changes rapidly, the accuracy decays rapidly (Jannink, 2010; Iwata et al., 2011; Yabe et al., 2014b). In the present study, prediction accuracy deteriorated with selection cycles, especially for traits with high prediction accuracy at GS1 (Figure 5). The change in the prediction ability of models caused inconsistency in selected plants between old and new prediction models (Supplementary Figure S1). To maintain prediction accuracy, we updated prediction models once a year and found that the population continued to improve during the six GS cycles. Simulation studies (Iwata et al., 2011; Yabe et al., 2014b) showed that updating prediction models to follow the changing pattern of LD and to capture genetic relationships among individuals will be essential in GS for allogamous crops with low LD levels. Our study could confirm this statement empirically.

GS is suggested to increase genetic gain per year mainly via the acceleration of breeding cycles (Asoro et al., 2013; Resende et al., 2014). The degree of genetic improvement per cycle was the same in one cycle of GS and PS in the selection experiment in the present study (Table 4, Figure 3). Our simulation studies (Yabe et al., 2013, 2014b) also suggested that genetic gain per unit time is more important than gain per cycle; however, in our 3-year selection, there was no significant difference between GS and PS (Table 4) despite an extra cycle per year in GS. One reason might be the strong inbreeding caused by GS cycles: of the 12 plants selected at GS5, 5 were derived from one seed parent, 5 were derived from another seed parent, and the remaining 2 shared their grandmother. Rutkoski et al. (2015) showed that GS decreased genetic variance and increased mean inbreeding more rapidly than did PS even with the same level of genetic improvement. The decrease in genetic variation in a breeding population would decrease GS prediction accuracy and prevent long-term genetic improvement (Jannink, 2010; Yabe et al., 2016). Thus, maintaining genetic variation in a breeding population would be necessary to attain long-term improvement in GS.

In this study, we evaluated the performance of GS and PS to improve the selection index. The performance of GS and PS in the selection index was different from that of our target trait (i.e., seed yield per unit area). This is because the cultivation condition was different between this study and the past field experiment. The previous data was used to build the selection index. Moreover, the relationship between the target trait and traits involved in the selection index can change under the repeated selection cycles. An experiment to measure the seed yield per unit area of the Post-PS and Post-GS populations is necessary to evaluate the absolute performance of GS and PS conducted in this study.

In conclusion, GS can improve the genetic ability of allogamous crops with mass selection, but some issues remain to be solved. Model updating is necessary for maintaining the accuracy of GS across selection cycles to overcome the low level and changing pattern of LD in breeding populations. The routine application of GS might still be difficult (Bernardo, 2016). It would be necessary to develop a guideline for GS breeding. Especially in mass selection, which is still used in the breeding of various allogamous crops, the acceleration of breeding cycles and the possibility of pollen control with GS will enhance the genetic gain per unit time (Yabe et al., 2013, 2014b). The present study may encourage application of GS to mass selection breeding in allogamous crops.

## Author contributions

All authors conceived, designed and performed the experiments, analyzed and interpreted data, and wrote the paper. SY and HI performed statistical analysis. HE, TK, and SN performed bioinformatic analysis. TH and RO conducted field experiments and contributed materials. MU, HE, TK, SN, and YY contributed reagents and performed marker genotyping.

### Conflict of interest statement

The authors declare that the research was conducted in the absence of any commercial or financial relationships that could be construed as a potential conflict of interest.
