# Supplementary material for: Potential of Genomic Selection in Mass Selection Breeding of an Allogamous Crop: An Empirical Study to Increase Yield of Common Buckwheat
Source: Front Plant Sci. 2018 Mar 21;9:276. doi: 10.3389/fpls.2018.00276 (PMC5871932; doi:10.3389/fpls.2018.00276)
Supplement: Supplementary file 1 [file Table1.PDF]

Supplementary Table S1. Process of GS and PS breeding conducted in the three-year selection trial.

Year	Date	Event	Population	Experiment			Note
				Cultivation	Laboratory	Analysis	
2011	7/26	GS1 & PS1	Initial population	Sowing			
	8/12 - 8/17	GS1	Initial population		DNA extraction		
	8/24	GS1 & PS1	Initial population	Pollination			
	8/31 - 12/8	GS1	Initial population		Sequence library construction		
	10/17	GS1 & PS1	Initial population	Phenotyping			
- 2012	12/2 – 3/16	GS1	Initial population		NextGenSeq	NextGenSeq	Illmina HiSeq2000
2012	5/6	GS1 & PS1	Initial population			Selection	After pollination
	5/29 - 5/30		Initial population		Design of microarray probes		15 samples
	5/14	GS2	Post-GS1	Sowing			
	5/31 - 6/4	GS2	Post-GS1		DNA extraction		
	6/6 - 6/26	GS2	Post-GS1		Microarray analysis		
	6/29	GS2	Post-GS1	Pollination		Selection	Before pollination
	8/6	GS3 & PS2	Post-GS2 & Post-PS1	Sowing			
	8/26 - 8/28	GS3	Post-GS2		DNA extraction		
	9/5	GS3 & PS2	Popst-GS2 & Post-PS1	Pollination			
	10/9	GS3 & PS2	Post-GS2 & Post-PS1	Phenotyping			
-2013	12/13 – 2/7	GS3	Post-GS2		Microarray analysis		
2013	2/21	GS3 & PS2	Post-GS2 & Post-PS1			Selection	After pollination
	4/4	GS4	Post-GS3	Sowing			
	4/23 - 4/24	GS4	Post-GS3		DNA extraction		
	4/28 - 5/23	GS4	Post-GS3		Microarray analysis		
	5/25 - 5/29	GS4	Post-GS3	Pollination		Selection	Before pollination
	8/7	GS5 & PS3	Post-GS4 & Post-PS2	Sowing			
	8/25 - 8/28	GS5 & PS3	Post-GS4 & Post-PS2		DNA extraction		
	9/10	GS5 & PS3	Post-GS4 & Post-PS2	Pollination			
	9/24 - 10/31	GS5 & PS3	Post-GS4		Microarray analysis		
	10/18	GS5 & PS3	Post-GS4 & Post-PS2	Phenotyping			
	11/4	GS5 & PS3	Post-GS4 & Post-PS2			Selection	After pollination
	11/6	GS6	Post-GS5	Sowing			
	11/23	GS6	Post-GS5		DNA extraction		
	11/25 – 12/17	GS6	Post-GS5		Microarray analysis		
-2014	12/17 - 1/4	GS6	Post-GS5	Pollination		Selection	Before pollination
