# Supplementary material for: Potential of Genomic Selection in Mass Selection Breeding of an Allogamous Crop: An Empirical Study to Increase Yield of Common Buckwheat
Source: Front Plant Sci. 2018 Mar 21;9:276. doi: 10.3389/fpls.2018.00276 (PMC5871932; doi:10.3389/fpls.2018.00276)
Supplement: Supplementary file 2 [file Table2.PDF]

Supplementary Table S2. Population mean (and standard deviation) measured in 2013 to evaluate the breeding schemes after two years of selection. The populations that do not belong to the same letter indicate a significant difference ($P < 0.05$) in their phenotypic values. For selection index, relative values when the value at the initial population is set as 100% are shown in the second bracket in each box.

	Main stem length (cm)	Number of nodes	Days to first flowering (days)	Number of flower clusters	Number of primary branches	1000 seed weight (g)	Test weight (g/l)	Number of secondary branches	Number of seeds in a plant	Selection index
**Post-GS1 & Post-PS1 (n=40)**	81.81 c<br>(16.89)	10.88 b<br>(1.86)	20.35 b<br>(1.86)	35.33 b<br>(26.76)	3.58 n.s.<br>(1.06)	28.10 a<br>(4.43)	493.77 n.s.<br>(82.74)	5.10 ab<br>(2.44)	209.86 b<br>(129.23)	23.79 b<br>(4.40)<br>(100.00%)
**Post-GS2 (n=39)**	88.45 bc<br>(16.62)	12.05 a<br>(1.73)	20.28 b<br>(1.93)	74.29 ab<br>(38.71)	3.69<br>(1.28)	26.78 ab<br>(3.37)	520.51<br>(71.86)	5.38 ab<br>(2.82)	291.72 ab<br>(177.80)	26.23 ab<br>(4.70)<br>(110.23%)
**Post-PS2 (n=35)**	91.22 bc<br>(18.20)	11.80 ab<br>(1.41)	20.51 ab<br>(2.99)	60.31 b<br>(29.59)	3.40<br>(1.46)	27.23 ab<br>(6.42)	537.78<br>(88.29)	4.63 b<br>(2.25)	272.14 ab<br>(145.80)	25.95 ab<br>(4.49)<br>(109.08%)
**Post-GS3 (n=37)**	97.19 ab<br>(20.95)	12.86 a<br>(1.97)	20.78 ab<br>(1.57)	90.54 a<br>(39.15)	3.97<br>(1.41)	25.47 b<br>(2.59)	528.65<br>(43.37)	6.68 a<br>(2.77)	362.24 a<br>(170.73)	28.21 a<br>(4.31)<br>(118.56%)
**Post-GS4 (n=33)**	106.11 a<br>(22.28)	11.91 ab<br>(2.39)	21.64 a<br>(1.75)	89.82 a<br>(48.17)	3.39<br>(0.97)	26.57 ab<br>(3.00)	533.36<br>(57.55)	5.73 ab<br>(1.79)	356.82 a<br>(178.03)	28.79 a<br>(4.98)<br>(120.99%)
