# Supplementary material for: Potential of Genomic Selection in Mass Selection Breeding of an Allogamous Crop: An Empirical Study to Increase Yield of Common Buckwheat
Source: Front Plant Sci. 2018 Mar 21;9:276. doi: 10.3389/fpls.2018.00276 (PMC5871932; doi:10.3389/fpls.2018.00276)
Supplement: Supplementary file 3 [file Image1.PDF]

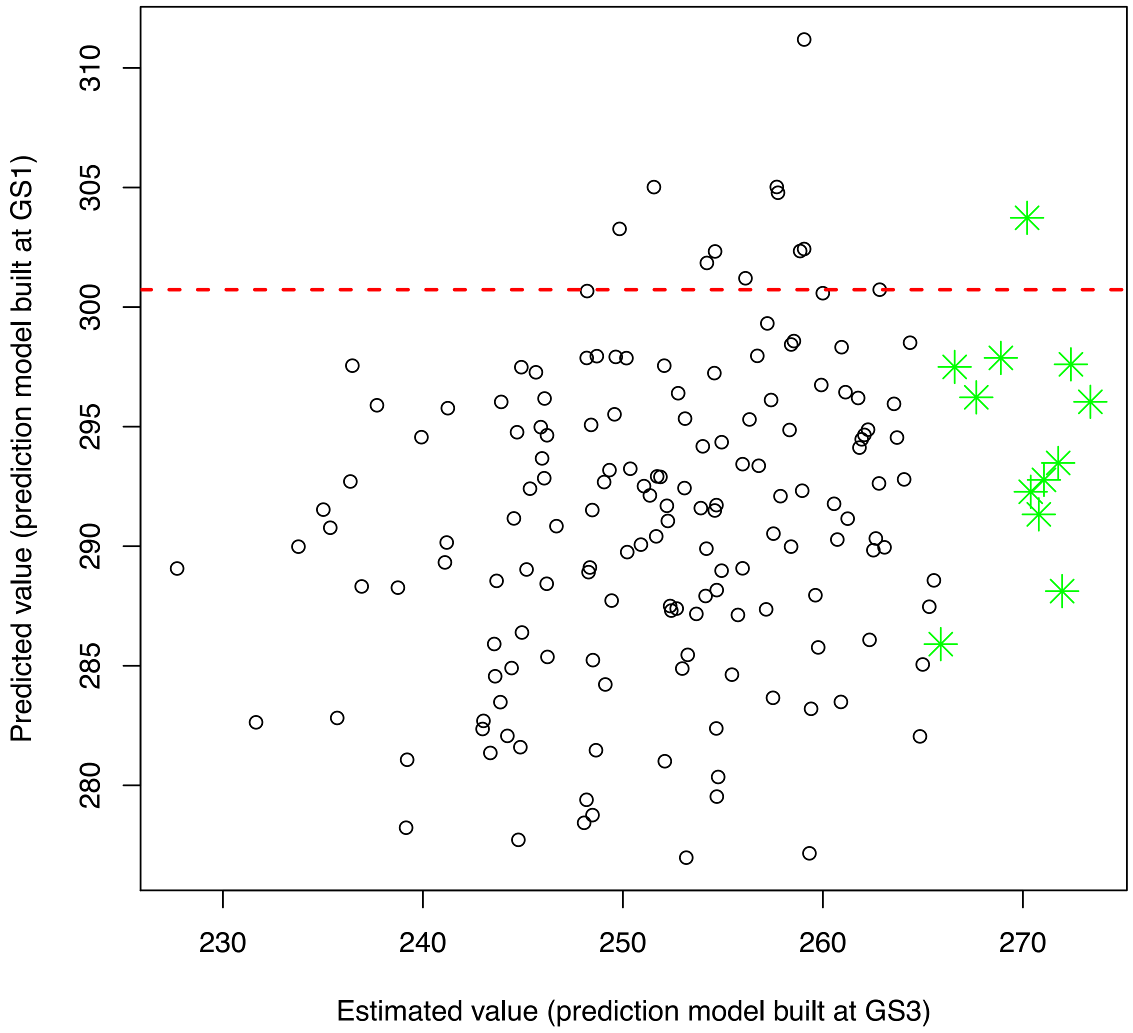

Supplementary Figure S1. The relationship between the estimated (i.e., values calculated using the updated model built at GS3) and predicted values (i.e., values calculated using the non-updated model built at GS1) of selection candidates at GS3. The red horizontal line represents the selection threshold when the non-updated model was used. Green asterisks represent the genotypes that were selected at GS3 based on the estimation by the updated model.
